# The Role of Title IX Coordinators on College and University Campuses

**DOI:** 10.3390/bs8040038

**Published:** 2018-04-05

**Authors:** Jacquelyn D. Wiersma-Mosley, James DiLoreto

**Affiliations:** School of Human Environmental Sciences, University of Arkansas, Fayetteville, AR 72701-1201, USA; diloreto@uark.edu

**Keywords:** Title IX coordinators, college campus, sexual violence

## Abstract

The purpose of this study was to better understand the role of Title IX coordinators and their policies across four-year universities and two-year community colleges in the United States (U.S.). There is little information regarding Title IX coordinators’ training, background, and policies on how they handle Title IX investigations regarding sexual violence. The data come from an online survey that included 692 Title IX coordinators across four-year (private and public) and two-year campuses and represented 42 different states in the US. The current study found that most Title IX coordinators were in part-time positions with less than three years of experience. Most of the coordinators and their investigators were trained in Title IX policies. Most coordinators provide Title IX training for their students and faculty, and most have completed a campus climate survey; however, 15% had not completed a survey. The findings suggest that the majority of campuses are continuing to increase their Title IX visibility; however, there are several recommendations for campuses to improve their policies. The current study was able to shed light on how Title IX coordinators do their jobs and the role they play in helping with the challenging issues surrounding sexual violence at institutions across the nation.

## 1. Introduction

Sexual violence against college women continues to be a pervasive public health issue with approximately one in five women experiencing sexual assault and one in nine women experiencing rape while in college [1,2,3]. In 2011, the Department of Education’s Office for Civil Rights (OCR) [4] issued a *Dear Colleague Letter* (*DCL*) [5] focused on the need to address sexual violence under Title IX of the Education Amendment of 1972 (Title IX) [6], which prohibits gender-based discrimination in educational programming or activities receiving federal funding. Specifically, the letter stated that “The sexual harassment of students, including sexual violence, interferes with students’ right to receive an education free from discrimination and, in the case of sexual violence, is a crime.” In response to these guidelines, the United States Department of Education began investigating Title IX violations. They have subsequently released a list of college campuses under investigation for mishandling or inappropriately handling cases of sexual violence in accordance with Title IX. The list, which started out with 55 campuses in 2014, has increased to 337 active investigations for possible mishandling of reports of sexual violence at 242 colleges as of 2018 (see the Chronicle of Higher Education Title IX Tracker [7]).

Originally established for the first time within OCR’s 2001 guidance document and again within the 2011 *DCL*, campuses must appoint a specific Title IX coordinator with the primary responsibility of coordinating campus compliance with Title IX, including grievance procedures for resolving Title IX complaints. The Title IX coordinator is responsible for coordinating responses to all complaints involving possible sex discrimination. This responsibility includes monitoring outcomes, identifying and addressing any patterns, and assessing effects on the campus climate. Such coordination can help campuses avoid Title IX violations, particularly violations involving sexual harassment and violence, by preventing incidents from recurring or becoming systemic problems that affect the wider community.

However, there is little, if any, empirical research that has examined the role of Title IX coordinators regarding how they handle Title IX complaints, their training, background, and their specific knowledge of campus resources and Title IX federal legislation. Title IX coordinators *directly* impact campus response to victims of sexual violence and, as the number of OCR investigations rises, are increasingly under pressure to manage this issue within institutions of higher education. The purpose of this study was to better understand the role of Title IX coordinators and their Title IX policies across four-year public/private universities and two-year community colleges in the United States (U.S.). A better understanding of these coordinators on campuses could assist in the effort to develop or improve current campuses and their Title IX policies and programming. It could also provide information that can assist campuses in understanding how to improve their Title IX presence to help victims to better understand the resources available to them. 

### 1.1. History of Title IX Guidelines

Title IX of the Education Amendments (1972) is a federal law that prohibits sex-based discrimination in higher education. In 1990, the Clery Act (also known as the Crime Awareness and Campus Security Act) [8] was amended to the federal financial aid laws to require all college campuses to disclose their campus crime statistics and security information. The Violence Against Women Act (VAWA) [9], established in 1994, provided legal definitions of domestic violence, dating violence, sexual assault, and stalking and was added to the crimes disclosed under the Clery Act [8].

In 2001, the Department of Education issued *Revised Harassment Guidance* [10], which first noted the legal obligation of schools to establish one individual on campus who would be responsible for all matters relating to Title IX compliance. This individual is required to have appropriate training on the law and the issues covered under it. In 2011, the Department of Education issued two letters regarding Title IX protections, including a new *Revised Sexual Harassment Guidance* [10], which reiterated that “schools are to ensure that employees are trained so that those with authority to address harassment know how to respond effectively”. ORC also issued the *Dear Colleague Letter* [5], which stated unequivocally that all education institutions, including college campuses (both public and private), as well as *K-12* secondary schools, must take immediate action to eliminate hostile environments, prevent recurrences, and address any effects. The letter also provided specific guidance procedures: campuses and schools must adopt and publish grievance procedures; employees must be trained to know how to report to officials (including teachers, law enforcement, administrators, counselors, general legal counsels, health personnel, and residential assistants (RAs)); polices must be published that include a notice of nondiscrimination; and all of these must be combined with education and training programs. In addition, their appointed Title IX coordinator must be easily accessible for all students and employees and must be included on a campus webpage that lists their name and contact information. And if more than one coordinator exists, campuses and schools must decide who will handle cases separately for students and faculty. The *DCL* also indicated that Title IX coordinators should not have any other job responsibilities that may create a conflict of interest (e.g., serving as general legal counsel). They must have adequate training on sexual harassment, sexual assault, and all grievance procedures that include equitable and prompt resolutions. Their resolutions can include voluntary mediation, but this was not strongly encouraged for sexual assault complaints. Lastly, “prompt time frames for major stages of the complaint process” (*DCL*, [5], p. 9) were defined as 60 calendar days following the receipt of a complaint.

According to OCR, there are certain rules campuses *should* abide by when dealing with Title IX issues. Not only is it difficult to determine whether campuses even abide by these rules, there is very little information about their entire processes given that campuses across the country operate differently. In terms of student conduct boards and appeal panels, there is no empirical research that examines who hears sexual assault cases and meets with victims and perpetrators. There is also little research that assesses whether Title IX coordinators are creating mandatory training sessions for those handling cases at the campus-level, especially given that one study discovered that approximately 30% of universities did not train the persons on the hearing panels [11].

### 1.2. Campus Responses

Prior to the 2011 guidance document published by OCR, Karjane, Fisher, and Cullen [12] assessed campus policies from 2438 institutions of higher education (IHE) and found that: 97% of campuses did not have anti-stalking measures in their school policies, only 46% had a separate sexual harassment policy; only 20% even mentioned harassment in their policies; 25% of schools did not provide a Title IX coordinators’ contact information; 40% of campuses were not training their students or faculty in regards to Title IX policies; and training was only mandatory in 34% of campuses. Following the 2011 updated guidelines and the *DCL* [5], Amar et al.’s study [13] included over 1000 campus administrators at four-year universities and found that most campuses were using hearing panels for their investigations that included students, faculty, staff, and administrators. However, 33% of those campuses reported a single decisionmaker, usually an individual within the Student Affairs department not associated with the case or an outside investigator. When respondents were found responsible for sexual misconduct, sanctions varied from suspension, expulsion, no-contact orders, counseling, community service hours, restitution, or fines.

Richards [14] conducted a follow-up study to Karjane et al. [12] with 820 IHEs, and found that 95% of campuses had a Title IX policy, 67% of campuses identified a Title IX coordinator, and 81% of campuses listed their policies and procedures regarding Title IX. However, the specific training and level of commitment of the Title IX coordinators were missing from these studies. Recently, Paul [15] conducted semi-structured interviews with 15 college campuses’ Title IX coordinators. Most coordinators reported that their jobs were a challenging ‘beast’ when describing the amount of work and responsibility of their Title IX position. Most wore ‘multiple hats’ in that they had dual roles/jobs on their campuses, with the majority working in either Student Affairs (33%) or Human Resources (27%) departments. Only 20% had more than four years of experience, while 80% had less than five years of experience. Most were appointed to their position or absorbed the role for various reasons; however, they were not relieved of any other duties and received no increase in their salary. One woman stated “that in order to shift the culture around sexual misconduct, there needed to be more investment in organizational learning than just naming a person” [15] (p. 66). Coordinators reported that their roles were emotionally charging and heavy at times, as they needed to know how to work the university political system in terms of getting resources and making things happen. They also needed to be well known by the campus community, which they found challenging. But the dominant theme that emerged from these 15 Title IX coordinators was that connecting to college faculty and training them on Title IX was the most challenging, as they were the “hardest nuts to crack” [15] (p. 80). Lastly, one coordinator stated: “The target keeps moving. The rules sometimes keep changing. The interpretation of things is changing. It’s not always black and white, so that’s one of the challenges” [15] (p. 78). Thus, most felt that it was difficult to keep up with changing regulations and the complex issues of Title IX. Time also seemed to be a huge issue; coordinators stated that it was difficult to close cases within 60 days, especially when they held other full-time jobs.

Federal guidelines remain a moving target for administrators; in September 2017, the newly installed republican Secretary of Education reversed many of the newly established policies within the 2011 *DCL* and the revised guidelines. She also reversed a 2001-established guideline, which strongly discouraged utilizing mediation as a means for adjudication. The current OCR ended the requirement that IHEs utilize the preponderance of evidence standard in adjudicating complaints [16]. Currently, the Department of Education has not released the promised revised guidelines. However, attorneys and advocates have indicated in media reports that a large percentage of colleges have ignored this guidance and are maintaining their policies based on the *DCL* authored by the previous administration [17].

In general, many four-year public and private colleges offer a variety of educational programs (e.g., rape awareness and prevention programs) and on- and off-campus services to victims (e.g., counseling, hotlines, and peer support) and have implemented security measures (e.g., card access to buildings and improved lighting) to address the risk of sexual victimization [18,19,20]. Some campuses have developed or improved sexual assault reporting procedures and investigative training of their public safety/law enforcement officers [21]. Most postsecondary institutions, particularly larger public and private colleges, also offer on-campus disciplinary procedures for sexual assault cases. Despite the emergence of concern about the sexual victimization of college students, little empirical research has been published about the Title IX process and the role of coordinators in addressing sexual violence on campus through prevention, support services, reporting, investigation, and adjudication.

### 1.3. The Current Study

One of the fastest growing fields within Student Affairs departments on college campuses is Title IX-related jobs [22]. The qualifications needed are substantial (listening skills, organization, and follow-through) with unpredictable hours, and they have to be extremely knowledgeable about sexual violence and Title IX, yet they must remain neutral and unbiased at all times. Typically their jobs are tacked onto other job duties, with no extra time or pay allotted [23]. The job is also stressful with no room for error as students are relying on them to make some of the most important decisions in students’ lives. As well, there are changing rules and regulations from OCR and Title IX policies of which coordinators and campuses must be aware, or they could be held accountable (i.e., OCR Title IX investigations). Using empirical data, the aim of this study was to use an exploratory design to understand the roles of a national sample of Title IX coordinators from two-year community colleges and four-year private and public universities. The purpose was to examine Title IX coordinators’ backgrounds, training, accessibility, sexual assault policies, and practices on their respective campuses. Understanding how campuses are responding to the ever-changing and often confusing guidelines could inform future policymakers seeking to prevent and adjudicate campus sexual violence.

## 2. Methods

### Procedures and Participants

According to a recent National Center for Education Statistics report [24], there were 17 million undergraduate students in the United States attending degree-granting postsecondary institutions, which are identified as institutions that are four- or two-year institutions and either public or private (for nonprofit). The focus was on these campuses (excluding those outside the geographical U.S.) with a student enrollment of at least 1000 students. Approximately 850 two-year community colleges and 1478 four-year public and private-nonprofit universities were identified via the Campus Safety and Security Data Analysis Cutting Tool provided by the Office of Postsecondary Education of the U.S. Department of Education [25]. First, using Google to search campus websites, a team of researchers (including the primary author, a graduate student, and undergraduate students) created a database that included campus Title IX coordinators’ names, emails, and other pertinent information that could be derived from campus websites. After collecting the list of names and emails, an online, close-ended, 54-question survey via Qualtrics was created and sent to the Title IX coordinators. The survey was completely anonymous with no identifying information regarding names or campus names to protect all individuals and their respective campuses. Participants were given an incentive opportunity where if they chose to participate by including their email address, one random winner would be chosen for a one-day, free training through the Association of Title IX Administrators (ATIXA) [26]. Institutional Review Board (IRB) was approved by the primary institution prior to data collection.

A wide range of descriptive information was documented about each campus website, such as whether a Title IX coordinator was listed (and if so, whether their name, their position/title, and email were available), whether a process for Title IX or sexual assault reporting was presented, and whether the website offered an online link for anonymous or confidential reporting for students. For the online survey, coordinators provided general demographic information, as well as answered questions regarding Title IX policies and procedures (i.e., reporting structures) on their campuses.

Researchers identified 850 two-year community colleges with student enrollment ranging from 1005 to 91,179 students. Of those, 785 campuses (92%) listed a Title IX coordinator email on their campus website; however, only 765 emails (90%) were able to be delivered due to emails bouncing. There were 1478 four-year universities identified (*n* = 614 public, *n* = 864 private-nonprofit) with student enrollment ranging from 1000 to 66,046 students. Approximately 1448 campuses (98%) listed a Title IX coordinator email on their campus website; however, only 1394 emails (94%) were delivered, due to some emails bouncing.

A total of 718 Title IX coordinators participated in the online survey; however, 26 were removed because the majority of their answers were missing. Thus, the final sample included 692 Title IX coordinators across four-year (private: *n* = 281; public: *n* = 198) and two-year campuses (*n* = 213) and represented 42 different states in the US, for a total response rate of 32%. Of the participants, 7% of their campuses were located in metropolis areas, 22% were located in urban areas, 32% were located in suburban areas, and 40% were located in rural or small-town areas. 

Of the 692 Title IX coordinators who participated in the survey, most identified as female (70%), and the average age was 48 (*SD* = 10.22), ranging from 25 to 78. Most coordinators (71%) identified as Caucasian/White, 16% as African American/Black, 4% as Hispanic/Latino, and 9% as mixed ethnicity or “other”. The majority (31%) reported that their salaries were between $100,000 and $149,999, while 29% reported $75,000–$99,999, 24% reported $50,000–$74,999, 12% reported $15,000–$49,999, and 4% did not disclose. As for education backgrounds, 47% reported having a master’s degree, 22% had a J.D., 20% had a Ph.D., 8% had a bachelor’s degree, and 3% reported “other”. The majority of the respondents (94%) reported that they were the primary Title IX coordinator; however, only 33% indicated that this was a full-time position. Some coordinators (18%) reported that they were also the investigator, and 11% reported additional duties (i.e., deputy coordinator or a mixture of these roles). The range of time spent in their Title IX role varied: 18% reported less than one year of experience, 25% had 1–2 years, 22% had 2–3 years, 19% had 3–5 years, 11% had 5–10 years, and 6% reported more than 10 years of experience. 

## 3. Results

### 3.1. Campus Websites

When researching two-year community college websites, 725 (out of 850; 85%) had a webpage with information regarding their Title IX process. Approximately 793 campuses (93%) listed the name of their Title IX coordinator, and 785 (92%) listed an email of the Title IX coordinator. As for website information regarding Title IX policies, 671 community colleges (79%) listed their policy, but only 101 community college campuses (12%) provided an online link for students to report an incident, and only 57 campuses (7%) provided an anonymous link.

When researching four-year university campuses, 1367 (out of 1478; 92%) had a webpage with information regarding their Title IX process. Approximately 1443 campuses (98%) listed the name of their Title IX coordinator, and 1448 (98%) listed an email of the Title IX coordinator. As for website information regarding Title IX policies, 1173 universities (79%) listed their policy, but only 348 universities (24%) provided an online link for students to report an incident, and only 206 universities (14%) provided an anonymous link. 

### 3.2. Online Survey

There were a wide range of academic roles that the Title IX coordinator was also involved in on their campus: 25% were in Human Resources departments, 16% in Diversity/Equity/Inclusion/EEO roles, 12% in Student Affairs departments as Dean or Director, 10% in Student Affairs departments as Vice President/Chancellor, 20% reported “other” (i.e., in Admissions, Affirmative Action, Student Activities, or Compliance departments or in general counsel, Clery Act coordinator, or advisor roles), and 17% were in a variety of other areas (i.e., Academic Affairs, Americans with Disabilities Act (ADA), Athletics, Compliance, Financial Operations, or the Office of the Provost). With respect to reporting structures on their campuses, the majority (48%) indicated that they report to the President or Chancellor, while 13% report to Student Affairs departments, 8% to the Vice President for Administrative Affairs or Business/Finance, 6% to Human Resources departments, 5% to general counsel/legal departments, 4% to Academic Dean/Provost, 3% to Equity/Inclusion departments, and 13% to “other”; and few (less than 5%) reported to multiple areas. As for their Title IX investigation methods, the majority (72%) reported using multiple methods: 52% use team investigation, 44% have panel adjudication/decisionmakers, 39% use a single investigator (who does not decide), 37% have appellate bodies or offices (when appeals are done), 23% use a single investigator/decisionmaker model, 21% have single decision makers, and 6% reported using another method. Approximately 202 Title IX coordinators reported that their appellate bodies were made up of various entities: administrators, faculty/staff, students, presidents, and provosts. 

When a Title IX complaint is received, coordinators reported that an average of 43% of their cases are resolved in adjudication, 18% of their cases are resolved in mediation, 37% are resolved in investigation without adjudication, and 34% are resolved in an informal method (not mediation). If a Title IX complaint required adjudication, the majority (37%) of decisions are made by the Title IX coordinator, while 14% are made by hearing panels (made up of students, faculty, staff, and administrators), 7% by the investigator, 5% by the hearing officer, and 17% reported “other” (such as a deputy or various other entities on campus). 

The 2011 *Dear Colleague Letter* [5] sought to establish prompt resolution for investigations by campus officials. Specifically, it states that it should not take longer than 60 days from start to finish. The average length of Title IX coordinators’ campus investigation lasted 48 days and ranged up to 270 days (*SD* = 30.52). Overall, of the 485 coordinators that responded to this question, 15% (*n* = 73) reported that the average case at their institution surpasses the 60-day threshold. Currently, while the new administration’s Department of Education drafts new guidelines, this threshold has been removed.

Title IX coordinators reported various organizations and memberships of which they were a part: 52% were affiliated with ATIXA [26], 13% with the Association for Student Conduct Administration (ASCA) [27], 23% with Student Affairs Administrators in Higher Education (NASPA) [28], 11% were Greek-affiliated alumni members, and 8% were former campus athletes. As for training, see Table 1 for a list of the various trainings that Title IX coordinators reported for themselves, investigators, and hearing panels, as well as which ones they felt were most helpful. The majority felt they were trained to do their job (88%) and reported that their investigators (82%) and hearing panels (64%) were also well trained.

As a means of ensuring that policies evolve to meet particular, campus-specific needs, the 2011 *DCL* [5] strongly recommends a campus climate survey. The majority of coordinators (57%) reported that they had completed a campus climate survey, 15% had not done so, and 28% were in the planning stages of conducting a survey. Nearly every Title IX coordinator surveyed (97%) provides Title IX training for their students and faculty, and of those, 63% of campuses require mandatory training for students and 78% for faculty.

Lastly, we examined whether there were differences among the three types of campuses (i.e., four-year private, four-year public, and two-year community colleges). First, the age (*F* = 4.17, *p* < 0.02) of the Title IX coordinators was significantly younger at four-year public universities (*M* = 45.91, *SD* = 9.90) compared to two-year community colleges (*M* = 48.84, *SD* = 9.69), but there were no differences in age compared to four-year private universities (*M* = 47.80, *SD* = 10.71). There were significantly fewer women in the role of Title IX coordinator at two-year community colleges compared to four-year universities (*x*^2^ = 14.64, *p* < 0.02). Title IX coordinators were less likely to be at full-time positions at two-year community colleges (18%) as compared to four-year private (39%) and four-year public (43%) campuses (*x*^2^ = 49.56, *p* < 0.001). There were no other differences among the three types of campuses in terms of demographics, reporting structures, or policies.

## 4. Discussion

The current study assessed the role of Title IX coordinators and their policies at 692 college campuses (including two-year community colleges, and four-year private and public universities) representing 42 different states (see Table 2). Similar to Paul’s [15] interviews with 15 Title IX coordinators, the current study found that most coordinators wore multiple hats, with 67% of them indicating that their Title IX role was *part*-time. Their university roles were primarily in Human Resources, Diversity/Equity/Inclusion/EEO, or Student Affairs departments or other various roles on campuses. And their role as Title IX coordinator was relatively recent, as 65% had less than three years of experience. This was in light of the fact that the majority of decisions were made by the Title IX coordinators when adjudication was required, which was on average 43% of the time. The burden placed on these part-time administrators is noteworthy. It is not surprising, therefore, that it took coordinators an average of 48 days to conduct the full investigation and decision-making process, which is under the 60-day limit by the *Dear Colleague Letter* [5]; however, the time taken to complete the Title IX processes ranged up to 270 days for some Title IX coordinators, which is concerning.

Due to the lack of knowledge regarding the training that Title IX coordinators receive, the current study assessed organizations and trainings in which they participated, as well as their investigators and hearing panels, if applicable. In general, the majority of Title IX coordinators felt that they were well-trained to do their jobs, along with their investigators, while there seemed to be some concern regarding hearing panels. When asked if they believed that their appellate bodies, regardless of their specific makeup, were adequately trained, 26% answered no. More confidence was shown in investigators and when self-reporting on their own training, with 61% of institutions having confidence in both. There is certainly room for improvement in this area. 

The majority of Title IX coordinators were current members of and trained with ATIXA [26], as were their investigators and hearing panels. ATIXA was noted as the most helpful of all of the training methods available for learning about the Title IX process. As noted on ATIXA’s webpage, their mission is to bring “campus and district Title IX coordinators and administrators into professional collaboration to explore best practices, share resources, and advance the worthy goal of gender equity in education”. ATIXA was founded by NCHERM (The National Center for Higher Education Risk Management [29]), which is the leading law firm with expertise in Title IX compliance, litigation, and expert witness services. Since 2010, ATIXA has provided a Title IX coordinator training and certification course, thus it is not surprising that it was the most commonly reported training resource used by campus Title IX coordinators. 

There was an increase from 40% of campuses [14] to 97% of campuses in the current study that reported providing Title IX training for their students and faculty; and the majority of those campuses stated it was mandatory training, which is certainly significant. The majority (57%) of Title IX coordinators in the current study reported that they had completed a campus climate survey. In addition, 28% of the current study’s coordinators were planning to conduct a survey, whereas 15% had not completed a survey. In a survey of 440 four-year public/private IHEs, McCaskill [30] found that only 16% of campuses had conducted an annual campus climate survey. However, this may have been due to timing (the report was only released in 2014), whereas now it has been several years, and surveys are much easier to distribute due to national companies that assist with campus climate surveys (e.g., Administrator Researcher Campus Climate Consortium (ARC3), Association of American Universities (AAU), and Higher Education Data Sharing Consortium (HEDS) [31,32,33]). Campus climate surveys are considered one of the most accurate ways to capture the rates of campus sexual assaults [34], yet they must also ensure student confidentiality and be short and electronically accessible with incentives to increase participation (e.g., only 7–53% of students responded to the AAU Climate Survey across 27 campuses [1]), as well as use variables that assess schools’ rates of sexual assault [35]. There is a lack of knowledge on the part of schools when they fail to survey their students. Schools that rely strictly on victim reporting, as some schools do, likely get relatively few reports in comparison to the sexual violence actually occurring [34]. Victims’ reasons for not reporting are dominated by beliefs that others, especially those in positions of authority, will not believe the victim [36,37,38]. According to the AAU survey [1], the primary reason for not reporting an incident was that the victim deemed it not important enough. Going forward, campuses should continue to increase the accessibility of policies, links, and Title IX coordinator information, along with campus climate surveys (by making these public surveys) and training of the university communities, which have all increased substantially in the last few years on college campuses. The increase of these preventative measures demonstrates at least some consistency with the spirit of the 2011 revised guidelines, which, as previously noted, are still utilized by a large contingent of institutions of higher education despite their recent revocation. 

The *Dear Colleague Letter* [5] encouraged institutions to value requests for confidentiality by stating that “the school should take all reasonable steps to investigate and respond to the complaint consistent with the request for confidentiality” (p. 5). While progress has also been shown in this area, only 24% of campuses currently provided an online link for students to report an incident, and a mere 14% provided an anonymous link. Karjane, Fisher, and Cullen [12] found that having this option is essential as it “allows student victims to come forward and talk to a trusted school official without the possibility of losing control of the process” (p. 93). 

The current study found that 216 Title IX coordinators resolved approximately one out of five (18%) of their cases with mediation. With the revocation of the 2011 *DCL*, mediation has now been deemed an appropriate way to resolve sexual assault allegations in the interim. This is a stark change from the guidance provided by the Office of Civil Rights under the last few administrations [4]. Mediation is not a productive means to adjudicate sexual assaults and could perpetuate already low reporting numbers. Koss, Wilgus, and Williamsen [39] explained that

“responses to sexual misconduct must acknowledge and obviate the negative effects of societal and individual norms that operate to silence victims and create opportunities for reabuse. When someone has been harmed by another person, mediation that provides neutrality and treats parties as equal partners in the resolution process is inappropriate”.

(p. 247)

As such, they argue that this is not an effective or appropriate way to combat campus sexual violence. 

The 2011 guidelines note that a Title IX coordinator should not have employment responsibilities that could be a conflict of interest with those of the Title IX coordinator; the document specifically notes that an example of this conflict would be an individual serving as both Title IX coordinator and general counsel. The current study found that this situation does not exist in the majority of campuses. However, there were several campuses who may have a conflict of interest because they use a dual Title IX coordinator/general counsel role, in addition to some coordinators who report directly to general counsel. The conflict of interest with such a structure could easily undermine the entire Title IX process on a campus.

Lastly, one would expect campuses to vary widely in their Title IX processes; however, the current study found that there were few differences among the four-year private and public and two-year community college campuses. It was not surprising that coordinators were less likely to be full-time at two-year community colleges, when it is likely that those campuses have less finances to fund a full-time Title IX coordinator, as compared to four-year universities, which may have more finances and higher student enrollment.

### 4.1. Limitations and Future Directions

These analyses provide important empirical insights to the roles of Title IX coordinators and their policies on college campuses in the US. Although the response rate was low (32%) for participation among 2100 possible Title IX coordinators, the current study did provide a national sample that included 692 coordinators/campuses from 42 different states. Even though the responses were anonymous, reporting such controversial and sensitive information could have resulted in social repercussions. Universities face a dilemma because being honest may help them to appear as though they want to end violence against women by encouraging reporting and drawing attention to the problem, but perhaps at the risk of gaining a reputation as a dangerous campus [34]. Lastly, the survey did not assess what was working in the Title IX processes on these campuses. Title IX processes and methods (e.g., hearing panels, single investigator, and appellate bodies) that work for one university may not work for another, and thus it is important for future research to assess what types of processes and policies are working more than others, in order to create a better process for both complainants and respondents. This could also help improve communication among all campuses, as there seems to be little, if any, collaboration of campuses regarding their Title IX policies and structures. Lastly, these findings may not only benefit college campuses, they may also overlap with *K-12* secondary educational policies and help assist Title IX coordinators working with high schoolers; very little is known about the sexual assault rates among high schoolers, as well as what OCR guidelines *K-12* schools are following, although recent research is addressing this gap [41].

Future research should examine the number of Title IX cases that occur on campuses, the outcome of those cases, the punishment (if any), and the demographics of the students involved. The Clery Act requires colleges that receive federal funding to disseminate an annual security report to the public every year, and it must include statistics of campus crime, plus details about efforts taken to improve campus safety. The primary purpose of requiring schools to report campus crime data is to provide students, faculty, and staff with an accurate portrayal of crime statistics. However, research [12,42,43] suggests that most universities *underreport* Clery statistics. However, a recent study done by Krebs et al. [44] concluded that their climate survey produced comparable results to Clery reports in rates of reported rape, which is promising. But for sexual violence to be included in Clery data, victims must know where to report; however, neither victims nor non-victims are likely to know the location of their school’s campus sexual assault center [45]. Although the current study found that Title IX coordinators and their contact information is indeed accessible via webpages, assessing whether students are actually aware of what Title IX is or what their campus coordinators do is clearly warranted. Thus, campuses should continue to educate students about Title IX, who their coordinators are, and how those Title IX coordinators can address student issues.

### 4.2. Conclusions

Overall, campuses are taking a diverse approach in their Title IX policies and adjudication, highlighting the challenges university administrators face. The Title IX coordinator position has become a critical and controversial figure in efforts to deal with sexual violence and its effects on campuses. While most regard the *DCL* [5] as being well-intentioned and one of the most comprehensive approaches to reducing campus sexual violence, the document has left much room for interpretation and, consequently, could lead to costly and protracted legal disputes [46]. This is evidenced by the large number of Title IX investigations coming from OCR and the news media. However, the work of a Title IX coordinator is not well understood by the public or even the campus community. It is important that Title IX coordinators be fully supported by the campus community, including administrators and faculty [15]. In general, Title IX coordinators need to be given full-time positions with additional resources to do their jobs better. The overwhelming pressure, changing policies, the unpopular position, and having to deliver hard news makes this one of the hardest and highest turnover jobs in the country [23]. The current study was able to shed light on how Title IX coordinators do their jobs and the role they play in helping with the challenging issues surrounding sexual violence at institutions across the nation.

## Figures and Tables

**Table 1:** Campus Title IX Coordinator Training.

	You (Title IX Coordinator)	Your Investigator (s)	Hearing Panel	Which Was Most Helpful?
ATIXA Investigator Training	282 (41%)	281 (41%)	58 (8%)	118 (17%)
ATIXA Title IX Coordinator Training	243 (35%)	54 (8%)	13 (2%)	102 (15%)
ASCA Sexual Misconduct Institute	67 (10%)	31 (5%)	10 (1%)	40 (6%)
ASCA Gehring Academy	39 (6%)	27 (4%)	4 (1%)	10 (1%)
ASCA Annual Conference	59 (9%)	23 (3%)	10 (1%)	10 (1%)
NCHERM	96 (14%)	53 (8%)	23 (3%)	18 (3%)
Local Law Enforcement Agency	128 (19%)	87 (13%)	21 (3%)	19 (3%)
NACUA	129 (19%)	44 (6%)	9 (1%)	42 (6%)
Academic Impressions	64 (9%)	32 (5%)	8 (1%)	13 (2%)
MAGNA Publications	62 (9%)	25 (4%)	7 (1%)	20 (3%)
Title IX Mastered	27 (4%)	16 (2%)	2 (3%)	10 (1%)
Other (*n* = 241)	173 (25%)	139 (20%)	87 (13%)	95 (14%)

*Note*: ATIXA = Association of Title IX Administrators; ASCA = Association for Student Conduct Administration; NCHERM = National Center for Higher Education Risk Management; NACUA = National Association of College and University Attorneys; MAGNA = Magna Publications: Higher Education Professional Development.

**Table 2:** A review of the literature on Title IX coordinators since the 2011 *Dear Colleague Letter.*

	Number of Participants	Hearing Panel Information	Policies on Title IX	One Coordinator Listed	Training Information	Dual Roles	Experience	Length of Investigations	Outcomes of Title IX Cases	Campus Climate Surveys
**Amar et al. (2014) [13]**	1067 IHEs	X			X	X			X	
**McCaskill (2014) [30]**	440 IHEs	X		X	X					X
**Konradi (2016) [40]**	Maryland IHE’s only	X			X					
**Richards (2016) [14]**	820 IHEs		X	X						
**Paul (2016) [15]**	15 Title IX Coordinators			X	X	X	X	X		
**Current Study**	692 Title IX Coordinators	X	X	X	X	X	X	X	X	X

*Note*: IHEs = Institutions of Higher Education.
